# Dynamic topological data analysis: a novel fractal dimension-based testing framework with application to brain signals

**DOI:** 10.3389/fninf.2024.1387400

**Published:** 2024-07-12

**Authors:** Anass B. El-Yaagoubi, Moo K. Chung, Hernando Ombao

**Affiliations:** ^1^Statistics Program, King Abdullah University of Science and Technology, Thuwal, Saudi Arabia; ^2^Department of Biostatistics & Medical Informatics, University of Wisconsin-Madison, Madison, WI, United States

**Keywords:** dynamic topological data analysis, time series analysis, fractal dimension-based testing, Higuchi fractal dimension, epileptic seizures

## Abstract

Topological data analysis (TDA) is increasingly recognized as a promising tool in the field of neuroscience, unveiling the underlying topological patterns within brain signals. However, most TDA related methods treat brain signals as if they were static, i.e., they ignore potential non-stationarities and irregularities in the statistical properties of the signals. In this study, we develop a novel fractal dimension-based testing approach that takes into account the dynamic topological properties of brain signals. By representing EEG brain signals as a sequence of Vietoris-Rips filtrations, our approach accommodates the inherent non-stationarities and irregularities of the signals. The application of our novel fractal dimension-based testing approach in analyzing dynamic topological patterns in EEG signals during an epileptic seizure episode exposes noteworthy alterations in total persistence across 0, 1, and 2-dimensional homology. These findings imply a more intricate influence of seizures on brain signals, extending beyond mere amplitude changes.

## 1 Introduction

Topological Data Analysis (TDA) is an emerging powerful framework for analyzing high-dimensional and noisy data by leveraging concepts from topology (Edelsbrunner et al., 2002; Carlsson et al., 2005). Within TDA, persistent homology stands out as a method for assessing the topological patterns within a filtration of simplicial complexes through varying spatial resolutions. Its core principle lies in quantifying the persistence (birth and death) of *k*−dimensional holes, where connected components represent 0-dimensional holes and circles or loops represent 1-dimensional holes, continuing to higher-dimensional holes as well, through the use of barcodes or other topological summaries (Carlsson et al., 2005; Bubenik, 2015; Adams et al., 2017). While TDA methods have faced criticism for perceived shortcomings in statistical inference (Chung and Ombao, 2021), the broad application of persistent homology to time series data has been demonstrated to be successful in various scientific fields ranging from the study of periodicity in gene expression (Perea et al., 2015), topological signs of financial crashes (Gidea and Katz, 2018) to medical imaging domains, including structural brain imaging, functional magnetic resonance imaging and electroencephalography (Stolz et al., 2017; Wang et al., 2019; Songdechakraiwut and Chung, 2020; El-Yaagoubi et al., 2023). Motivated by these advancements and their potential to uncover novel topological characterizations of the brain, this paper explores the dynamics of different topological patterns in brain signals, with a specific focus on epileptic seizures. Unlike the existing approaches that analyze resting-state fMRI data (Songdechakraiwut and Chung, 2020), our research delves into the intricate dynamics of EEG signals during seizure time, aiming to uncover deeper insights into the complex behavior exhibited by the brain during epileptic activity.

To capture the dynamic and non-stationary characteristics of brain signals, we employ a sliding window approach for encoding multivariate EEG signals as time-varying Vietoris-Rips filtrations. The assessment of topological information at each temporal point is facilitated by total persistence (TP), which represents the sum of persistence over all features in the *k* homology group of interest, expressed as $TP_{k}=\underset{\left(b_{i},d_{i}\right)\in H_{k}}{\sum }\left(d_{i}-b_{i}\right)$. Therefore, the time-varying total persistence serves as a topological summary of the evolving Vietoris-Rips filtrations, providing insights into the dynamics of brain activity as the patient enters into an epileptic state. To assess the statistical significance of the observed changes in total persistence, we propose a novel testing framework based on fractal dimension, enabling a robust evaluation of the altered topological characteristics. A visual inspection indicates that the EEG signal has increased variance (larger wave amplitudes) during seizure. Moreover, our proposed dynamic TDA method was able to detect significant changes in total persistence during the epileptic seizure across all homology dimensions (0, 1, and 2), indicating a more complex behavior in brain signals beyond a mere change in signal amplitude (or variance). Our findings shed new light on the intricate dynamics of brain connectivity during seizure. In Section 2, we provide a succinct overview of dynamic topological data analysis where we assume that the topological patterns under scrutiny are no longer static in time but rather dynamic. In Section 3, we recall the definition of fractal dimension alongside a few examples. Subsequently, we introduce a novel inference framework to evaluate the statistical significance of alterations in topological summaries based on this notion of fractal dimension. In Section 4, we present a simulation study that serves to illustrate the application of our approach using simulated data. In Section 5, we employ dynamic-TDA on EEG signals obtained from an epileptic subject and employ our proposed testing framework to assess the statistical significance of the changes in the topological structure induced by seizure. Our results provide compelling evidence that the seizure has a profound impact on the topological patterns manifested in the EEG signal. We have shared our analysis code in the GitHub repository: Dynamic-TDA. The repository includes two detailed Jupyter notebooks: one for our simulation studies and another for applying our method to real EEG data. We hope this resource will be valuable to researchers and practitioners.

## 2 Dynamic TDA

In recent decades, significant strides have been taken in the exploration of point cloud data. Approaches like t-distributed Stochastic Neighbor Embedding (t-SNE) and Uniform Manifold Approximation and Projection (UMAP) have emerged as powerful tools, demonstrating remarkable success in comprehending and visualizing intricate, high-dimensional datasets (Maaten and Hinton, 2008; McInnes et al., 2018). In contrast, the utilization of Topological Data Analysis (TDA), specifically through persistence homology, has yielded novel insights into point cloud data. By capturing the topological features of the ambient space at varying spatial resolutions, persistence homology offers a multiscale representation of the data that remains invariant under continuous deformations—a crucial characteristic for robust analysis (Edelsbrunner et al., 2002; Chazal and Michel, 2021).

**Figure 1:**
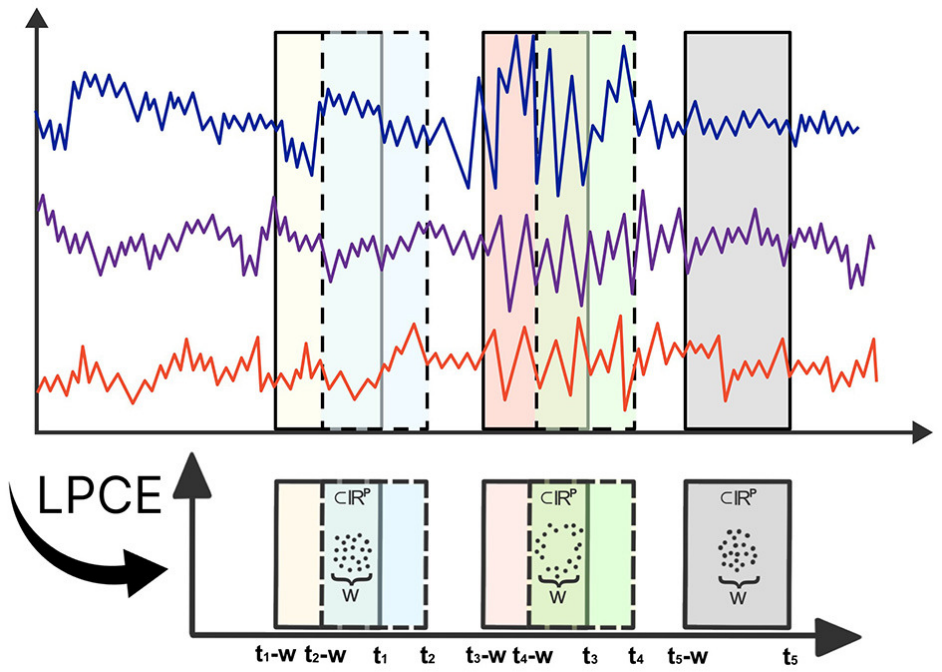
Illustration of Local Point Cloud Embedding (LPCE). Each *t*_*i*_ marks the ending time of the sliding window, and *w* denotes the width of each window.

**Figure 2:**
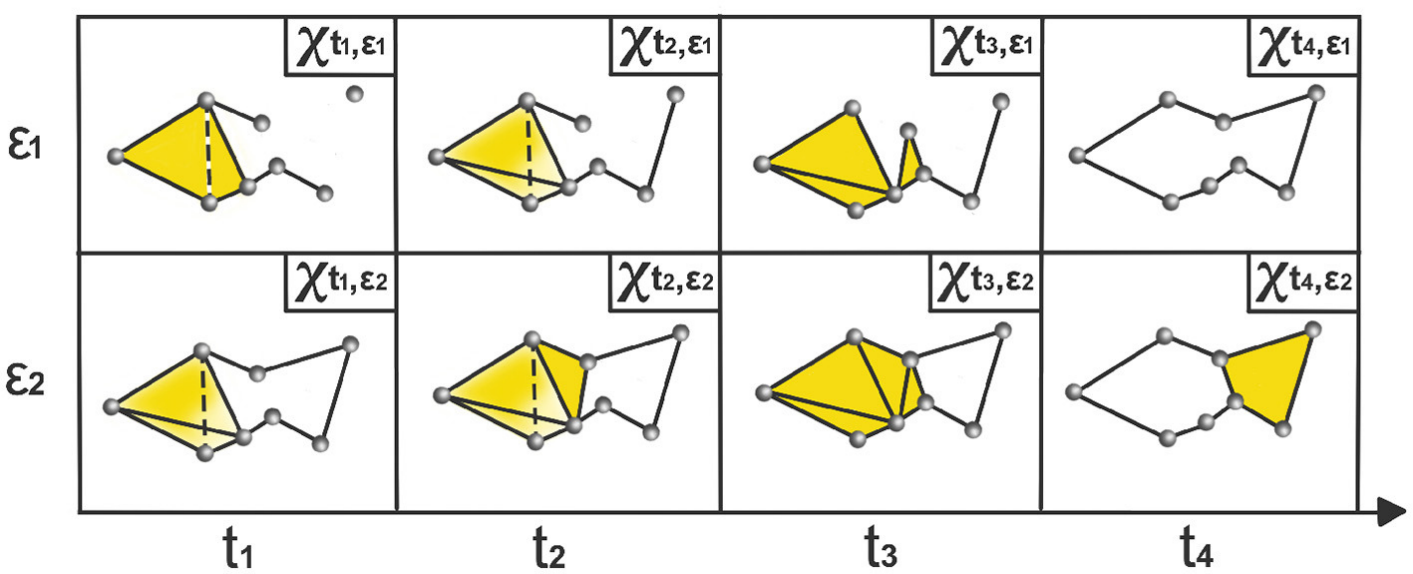
Time-varying Vietoris-Rips complexes based on LPCE. At each time point *t*_*i*_, the nodes represent observations within the sliding window starting at *t*_*i*_−ω and ending at *t*_*i*_. The parameter ϵ_*i*_ indicates the spatial resolution at which edges, faces, and higher-dimensional simplices are added.

Topological data analysis has made substantial contributions to the analysis of multivariate time series data (Gholizadeh and Zadrozny, 2018; El-Yaagoubi et al., 2023). Theoretical milestones, such as Takens' Theorem (Takens, 1981), offer practitioners a powerful framework to transform multivariate time series data in order to capture the topology of the underlying system dynamics using point cloud embeddings. These embeddings allow full use of TDA, facilitating a thorough investigation of the topological features present in the underlying dynamic system (Gholizadeh and Zadrozny, 2018; Gidea and Katz, 2018). To formalize these ideas, denote $X\left(t\right)={\left[X_{1}\left(t\right),\ldots ,X_{P}\left(t\right)\right]}^{\prime }\in {\mathbb{R}}^{P}$ to be a *P*-dimensional multivariate time series. Consider the time-localized point cloud embedding (LPCE), which involves a sliding window subset of the data with a length of *w* at time *t*, represented as *LPCE*(*t*) = [*X*(*t*), *X*(*t*−1), …, *X*(*t*−*w*+1)] ∈ ℝ^*P*×*w*^, which can be visualized in Figure 1. In contrast to the sliding windows and 1-persistence scoring (SW1PerS) approach introduced by Perea et al. (2015), our method relies on dynamic embedding and effectively handles multivariate time series data. Utilizing this LPCE, we construct the time-varying Vietoris-Rips filtration in Equation 1.


(1) $$\begin{array}{l} X_{t,\epsilon_{0}}\subset X_{t,\epsilon_{1}}\subset \ldots \subset X_{t,\epsilon_{n}} \end{array}$$

where $X_{t,\epsilon }$ is a time-varying simplicial complex, i.e., a combination of *k*-simplices, see example in Figure 2, where each *k*-simplex corresponds to (*k*+1)-tuples of brain channels that are pairwise within a distance ϵ at time *t*. When ϵ is sufficiently small, the complex consists only of individual nodes, and for sufficiently large ϵ, the complex becomes a single connected (*P*−1)-dimensional simplex.

For non-stationary signals, such as electroencephalograms (EEG), the sliding window approach is commonly used to analyze dynamic properties Möller et al. (2001); Antonacci et al. (2023). However, this method can introduce autocorrelations in the estimates and may not effectively handle abrupt model changes. To address these issues, our study employs non-overlapping windows. We select a window size that is small enough to capture abrupt changes, yet large enough to accurately define the shape of the point cloud.

The goal of persistent homology is to identify topological features such as *k*-dimensional holes that persist throughout the range of the parameter ϵ. The intervals of the form [ϵ_*b*_, ϵ_*d*_ are the lifetimes of *k*-dimensional holes that appear in the Vietoris-Rips filtration, which represent the critical topological information, that is usually encoded in barcodes (*B*_*k*_) or persistence diagrams (*PD*_*k*_). Following the approach in Bubenik (2015) and Songdechakraiwut and Chung (2020), we formally define the time-varying total persistence of the *k*-dimensional holes as the sum of all persistence values of *k*-dimensional holes at time *t*:


(2) $$\begin{array}{l} {\text{tv-TP}}_{k}\left(t\right)=\underset{\left[\epsilon_{b},\epsilon_{d}\right]\in B_{k}}{\sum }\left(\epsilon_{d}-\epsilon_{b}\right) \end{array}$$

these time series measure the total lifetime of all the *k*-dimensional holes at time *t*. A larger value indicates an extended persistence of topological features, signifying more robust and persistent structures in the data. Conversely, smaller values suggest more transient topological patterns.

## 3 A fractal dimension-based testing approach

In this section, we develop a novel statistical testing framework, based on the notion of fractal dimension, to evaluate the significance of structural breaks in total persistence functions. The proposed approach is quite general and can be applied in various contexts, particularly to assess structural changes in the mean functions utilizing the Cumulative Sum (CUSUM) approach. By employing this general testing procedure, our proposed approach enables researchers to effectively analyze and quantify structural changes, enhancing the robustness and interpretability of results across different settings.

### 3.1 Fractal dimension

Fractals and fractal dimension has a long history dating back to the 17-th century. However, our use of the term fractal is due to the pioneering work of mathematician Benoit Mandelbrot in the 1960s and 1970s (Mandelbrot, 1967, 1975). The existence of intricate, non-Euclidean geometries in various natural phenomena, such as coastlines, clouds, and ice crystals can be assessed by various measures. Fractal dimension, a fundamental concept in this field, serves as a measure of the complexity and degree of self-similarity exhibited by sets. It quantifies the relative change in the level of detail within a structure or object (e.g., length, area, and volume) in response to changes in the observation scale.

**Figure 3:**
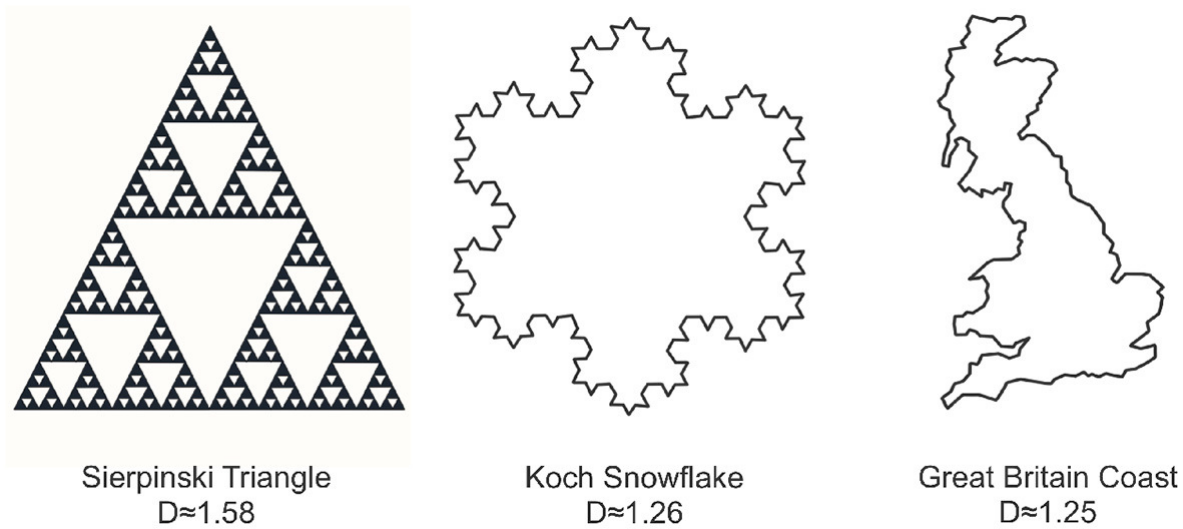
Three examples illustrating various forms of fractal behavior: the Sierpinski triangle **(left)**, the Koch snowflake **(center)**, and the coastline of Great Britain **(right)**. Each object is accompanied by its corresponding box counting fractal dimension.

Fractal sets are defined to be sets that exhibit self-similar patterns repeating at various scales. We can create some of these patterns mathematically, as seen in the Sierpinski Triangle and the Koch Snowflake in Figure 3. The Sierpinski Triangle builds recursively, revealing smaller triangles removed from its center in each step. The Koch Snowflake forms a fractal curve by replacing straight lines with smaller equilateral triangles. Moving beyond mathematical constructions, nature provides abundant examples, including the intricate coastline of Great Britain in Figure 3 and various other patterns found in trees, ferns, cauliflower, crystals, lightning, and more. These diverse examples, both mathematical and from nature, underscore the ubiquity of fractal structures in the world around us. Unlike the classical notion of Euclidean dimension, which is a positive integer, fractal dimension is not constrained to be positive integer-valued, as shown in the examples of Figure 3. One commonly used method to compute the fractal dimension is through the concept of box-counting (Mandelbrot, 1982; Iannaccone and Khokha, 1996; Gonzato et al., 2000).

In this approach, to compute the fractal dimension of a set *S*, it needs to be covered by a grid of boxes of a given size *r*, then the number of boxes *N*_*r*_(*S*) required to cover the set *S* is computed at each scale *r*. The fractal dimension is then calculated as the limit of the logarithm of the number of boxes needed to cover the set divided by the logarithm of the box size inverse, as the box size tends to zero. Mathematically, the fractal dimension *D* of a set *S* is given by:


$$D=\underset{r\to 0}{\lim}\frac{\log\left(N_{r}\left(S\right)\right)}{\log\left(1/r\right)}$$

where *N*_*r*_(*S*) is the number of boxes needed to cover the set *S* at scale *r*. This is visually demonstrated in Figure 3 which reveals notable distinctions in the fractal dimensions of the depicted objects. Specifically, the Sierpinski triangle exhibits the highest fractal dimension, occupying a substantial portion of the plane. In comparison, the Koch snowflake possesses a lower fractal dimension than the Sierpinski triangle, while the coast of Britain exhibits the lowest fractal dimension, occupying a comparatively smaller area in the plane.

### 3.2 Higuchi fractal dimension

Extending the concept of fractal dimension to the realm of time series analysis, Higuchi proposed a method for computing the fractal dimension of a time series based on a notion of curve length (Higuchi, 1988). The Higuchi fractal dimension (*HFD*) is determined by analyzing the relationship of the time series curve length, denoted by *L*(*k*), with the scale *k*. This method operates on the principle that, as the scale *k* varies, a smooth curve would exhibit a proportional variation in curve length, resulting in a *HFD* value closer to 1. In contrast, a more irregular or complex curve would yield a higher *HFD* value. Essentially, the *HFD* provides a quantitative measure of the irregularity or complexity inherent in a time series, offering insights into the underlying dynamics and patterns at different scales.

For a univariate time series *X*(*t*) observed at times *t* = 1, …, *T*, the curve length computation involves summing the absolute differences between consecutive observations that are lag *k* time units apart, as shown in Equation 3. This is typically computed for a range of scales, from 1 to *k*_max_, where *k*_max_ ≥ 2. At each scale, an average over *m* is considered, as in Equation 4.


(3) $$\begin{array}{l} L_{m}\left(k\right)=\frac{T-1}{\left\lfloor \frac{T-m}{k}\right\rfloor \times k^{2}}\overset{\left\lfloor \frac{T-m}{k}\right\rfloor }{\underset{i=1}{\sum }}|X\left(m+ik\right)-X\left(m+\left(i-1\right)k\right)|, \end{array}$$

(4) $$\begin{array}{l} L\left(k\right)=\frac{1}{k}\overset{k}{\underset{m=1}{\sum }}L_{m}\left(k\right). \end{array}$$

The HFD of the observed time series *X*(*t*) is then approximated by finding the slope of the best-fitting linear function through the data points $\{(\log\left(\frac{1}{k}\right),\log L\left(k\right)){\}}_{k=1}^{k_{max}}$. In other words, the curve length scale relationship follows Equation 5.


(5) $$\begin{array}{l} L\left(k\right)\propto k^{-HFD} \end{array}$$

Since its inception in 1988, the Higuchi Fractal Dimension (*HFD*) has emerged as a robust measure for quantifying the complexity and irregularity exhibited by one-dimensional time series signals. To assess the complexity of a time series, *HFD* evaluates how the curve length varies with respect to the scale parameter. If a time series displays self-repeating patterns, *HFD* tends to be larger than one. Conversely, for time series lacking self-repeating patterns, such as smooth curves (where zooming in removes motifs, reducing the curve to a straight line regardless of the initial shape), *HFD* tends to be closer to one. The *HFD* thus serves as a quantitative measure to assess the complexity of a time series by discerning the presence or absence of self-repeating motifs. For instance, in the realm of finance, the *HFD* has proven valuable for assessing the complexity of stock exchanges by analyzing the closing price indices (Rani and Jayalalitha, 2016). In the domain of physiological analysis, particularly in the evaluation of heart rate variability (HRV) to gauge autonomic nervous system (ANS) activity among controls vs. diabetic patients, the *HFD* has exhibited discriminative capabilities. Notably, when comparing healthy subjects to diabetic patients, the *HFD* proved to be significantly higher in individuals with diabetes (Gomolka et al., 2018). In the field of neuroscience, the *HFD* has been employed to examine the complexity of various brain signals, including electroencephalography (EEG) recordings (Nobukawa et al., 2019; Gladun, 2020). These examples highlight the broad spectrum of applications and the effectiveness of the *HFD* in capturing intricate patterns and irregularities inherent in time series data, such as abrupt shifts, oscillations, and other complex variations.

### 3.3 Higuchi fractal dimension and random walks

Analyzing multivariate time series dynamics is essential for understanding various natural and economic phenomena. By quantifying how a time series evolves over time, researchers can reveal underlying patterns and dependencies that may not be apparent at first sight. A key aspect of this analysis involves examining the autocorrelation and memory of the series, which are fundamental to informed predictions and effective modeling strategies. Within this framework, the Hurst exponent (*H*) serves as a pivotal measure, offering insights into the mean-reverting or trending behavior of the time series. It assesses the likelihood of a series to either maintain a consistent trend or revert to its mean. Higher values of *H* (approaching 1) suggest a persistent trend, indicating smoother, less volatile behavior, while lower values (approaching 0) indicate a more volatile and erratic series characterized by frequent mean reversions.

**Figure 4:**
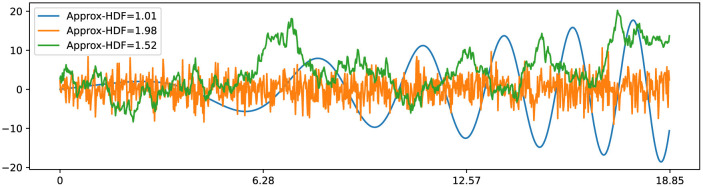
Three time series examples with corresponding Higuchi fractal dimension: $\text{smooth time series }x(t)=t\cdot \text{cos}({\text{t}}^{\text{2}}/10)$ has HFD ≈ 1; Gaussian white noise with σ=3 has HFD ≈ 2; standard Brownian motion has HFD ≈ 1.5.

In his study of Brownian motion, Benoit Mandelbrot established a remarkable connection between the Hurst exponent and fractal dimension, which paved the way for the development of fractal Brownian motion (fBm) (Mandelbrot and Van Ness, 1968). Subsequently, the relationship between the fractal dimension (also referred to as Hausdorff dimension) of fBm and the Hurst exponent was further elucidated in subsequent studies (Orey, 1970; Mandelbrot, 1982). This connection reveals that classical Brownian motion, represented by $B\left(t\right)=\int_{0}^{t}dW\left(t\right)$, possesses a fractal dimension of 1.5 (*HFD* = 1.5) and a corresponding Hurst exponent of 0.5 (*H* = 0.5). Here, *dW*(*t*) represents a normally distributed independent increment at each time step with *Var*[*dW*(*t*)] = *dt* and *E*[*dW*(*t*)] = 0. This relationship is expressed as *HFD* = 2−*H*, as depicted in Figure 4. Additionally, the observation holds for smooth curves, such as the *x*(*t*) = *t*·cos(*t*^2^/10), with a fractal dimension close to 1 (*HFD* ≈1) along with a Hurst exponent close to 1 (*H* ≈ 1). These insights into the fractal dimension of Brownian motion and smooth curves play a pivotal role in the development of our novel fractal testing methodology.

### 3.4 Fractal dimension and the CUSUM approach

The CUSUM method, a statistical tool widely used for detecting structural breaks or alterations in univariate signals, calculates cumulative sums of deviations from an expected or reference value over time (Page, 1954). Our objective is to devise a CUSUM-based approach specifically tailored for evaluating the presence of structural breaks in time-varying total persistence curves. These curves encapsulate meaningful information by dynamically assessing the shape of brain signals as they transition into epileptic states. Harnessing the expected random walk behavior of the CUSUM test statistic, which is anticipated to exhibit a fractal dimension of 1.5 under the assumption of no structural breaks, we will interpret deviations from this value as indicative of topological changes in brain signals.

Let *TP*_0_(*t*), *TP*_1_(*t*), and *TP*_2_(*t*) be the observed time-varying total persistence curves as defined in Equation 2. Then, define the cumulative sum of deviations as in Equation 6.


(6) $$\begin{array}{l} S_{k}\left(0\right)=0,\\ S_{k}\left(t\right)=S_{k}\left(t-1\right)+D_{k}\left(t\right) \end{array}$$

Let the null hypothesis *H*_0_ be defined as in Equation 7.


(7) $$\begin{array}{l} H_{0}:\forall t,𝔼\left[D_{k}\left(t\right)\right]=0. \end{array}$$

This null hypothesis posits that the expected value of deviations (*D*_*k*_(*t*)) is always zero, indicating no substantial changes in the *k*-dimensional topological features over time. To conduct a formal statistical inference under the aforementioned hypothesis, it becomes imperative to define a suitable test statistic and determine its reference distribution. The null hypothesis posits the absence of structural breaks, suggesting that deviations from the mean of total persistence *D*_*k*_(*t*) fluctuate around zero. This implies that observed deviations from the mean are viewed as random fluctuations, rather than suggestive of systematic changes in the underlying structure or dynamics.

Therefore, under the null hypothesis, we can exchange the observed deviations, producing a set of permuted deviations denoted as $D_{k}^{*}\left(t\right)$. The permutation process provides a reference distribution under the assumption of no systematic changes, reinforcing the idea that any observed deviations could occur randomly if there are no structural breaks. Subsequently, the cumulative sum of these permuted deviations, denoted as $S_{k}^{*}\left(t\right)$, is expected to exhibit characteristics reminiscent of a random walk. This expectation stems from the concept that, under the null hypothesis, the cumulative sum should demonstrate random and unpredictable behavior over time. Thus, the entire process aligns with standard practices in hypothesis testing, leveraging random permutation and cumulative sums to assess the significance of observed deviations under the assumption of no structural breaks.

In order to quantify the extent to which the observed sum of deviations *S*_*k*_(*t*) deviates from a random walk under the null hypothesis, reflecting the absence of structural breaks in total persistence, we utilize the *HFD* as defined in Section 3.2. By employing the *HFD*, it becomes possible to assess the dissimilarity between the behavior of *S*_*k*_(*t*) and that of a random walk. Specifically, the proximity of the *HFD* to 1 (or deviation from 1.5), indicates a departure from random behavior, thereby implying the presence of a structural break in the time-varying total persistence.

## 4 Simulations

The primary objective of this section is to demonstrate the efficacy of our approach in a simulated environment that replicates the time-varying topological properties of a multivariate time series. By conducting comprehensive simulation studies, we aim to validate the robustness and potential of our methodology under different conditions. We propose two examples to illustrate this: the first involves simple circular and spherical patterns, while the second features more complex topological structures with multiple cycles and voids. These scenarios are designed to test the adaptability and precision of our approach in capturing the dynamic topological features of the data.

### 4.1 First example: simple patterns

**Figure 5:**
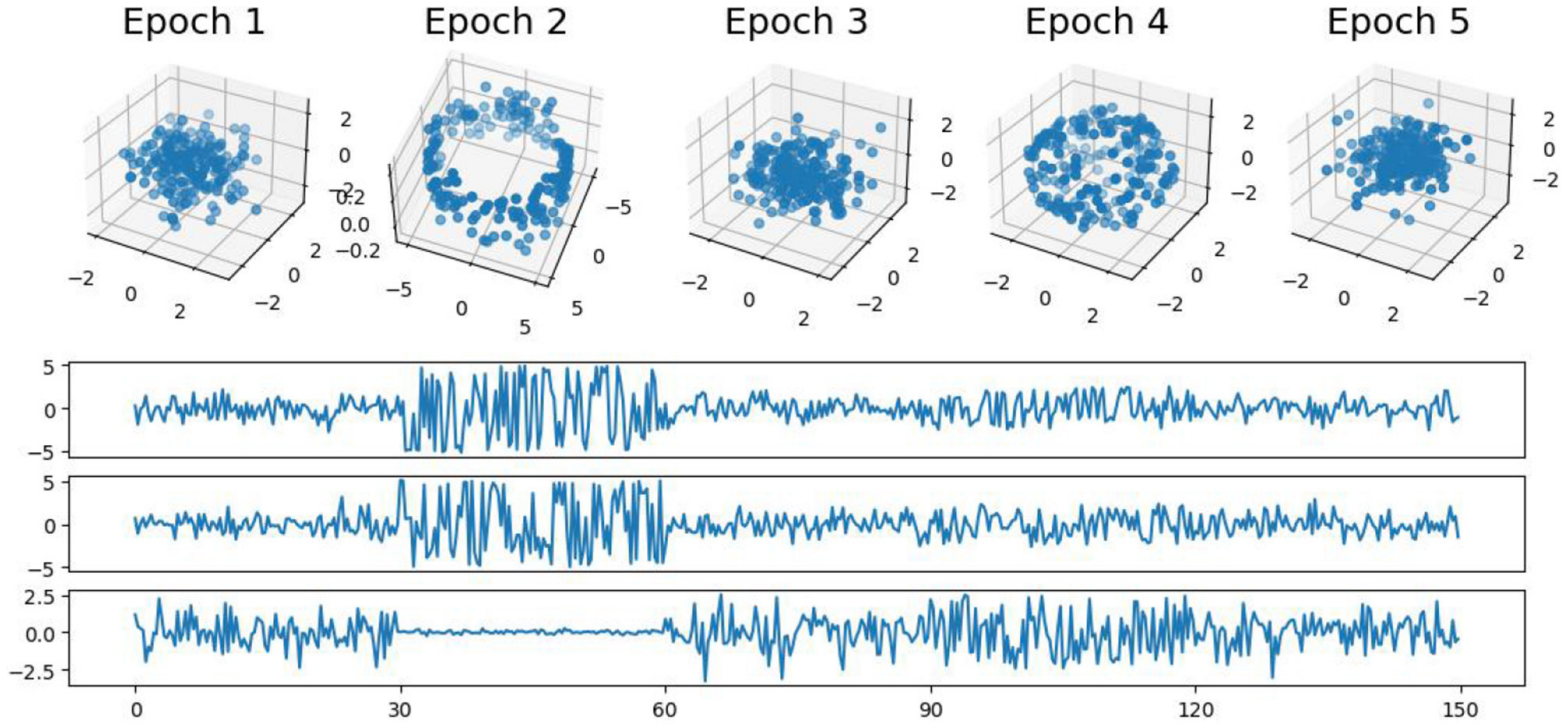
Simulated multivariate time series **(bottom)** with cloud point representation **(top)**. The second epoch exhibits a circular shape, while the fourth epoch takes on a spherical form. The first, third, and fifth epochs exhibit Gaussian uncorrelated random noise.

**Figure 6:**
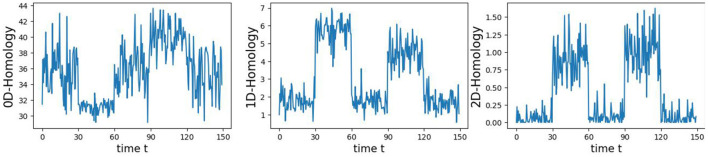
Estimated time-varying persistence curves reveal prominent peaks during the second (30–60 seconds) and fourth (90–120 seconds) epochs in both 1D and 2D-Homology, aligning with the expected circular and spherical shapes in the simulation.

In this first example, we generate a 3-dimensional time series consisting of 15,000 observations, equivalent to 150 seconds of data captured at a rate of 100 observations per second (see Figure 5). During the first, third, and fifth 30-second epochs, the observations are drawn from a 3-dimensional uncorrelated Gaussian distribution with a mean of zero and a standard deviation of 1. In the second 30-second epoch, the observations are drawn from a circle with radius of size 4, and in the fourth 30-second epoch, they are drawn form a sphere with radius of size 2.5. A visual representation of samples from this simulated data can be observed in Figure 5. Following the approach presented in Section 2, we employ a sliding window of size 50 and compute the total persistence to evaluate the dynamics of the topological patterns within the data. The estimated time-varying total persistence, as illustrated in Figure 6, provides a clear depiction of the evolving topology over time. Notably, an increase in the total persistence of 1D-Homology signifies the presence of holes in the cloud point, indicating a non-linear relationship among the components of the multivariate time series. Similarly, increase in the total persistence of 2D-Homology suggests the existence of cavities in the cloud point, reflecting interdependence among the time series components, now constrained to follow a non-linear relationship with a spherical shape. Conversely, a decrease in total persistence signals the disappearance of these topological patterns, leaving points randomly distributed in space.

The time-varying total persistence curves indicate an increase in total persistence during the second and fourth 30-second epochs for both the 1- and 2-dimensional features. Following the approached described in Section 3, we propose Algorithm 1 to assess the statistical significance of structural breaks in total persistence.

**Algorithm 1:**
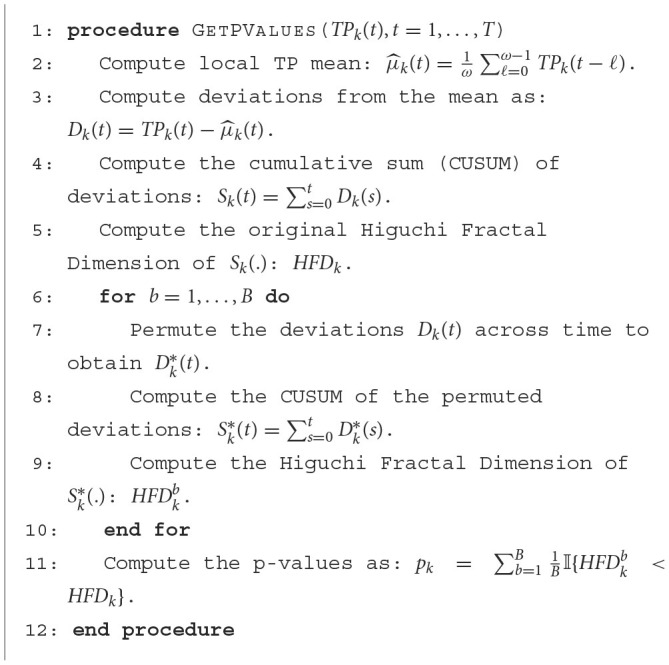
Computation of *p*-values based on the Higuchi Fractal Dimension of CUSUM of total persistence deviations.

**Figure 7:**
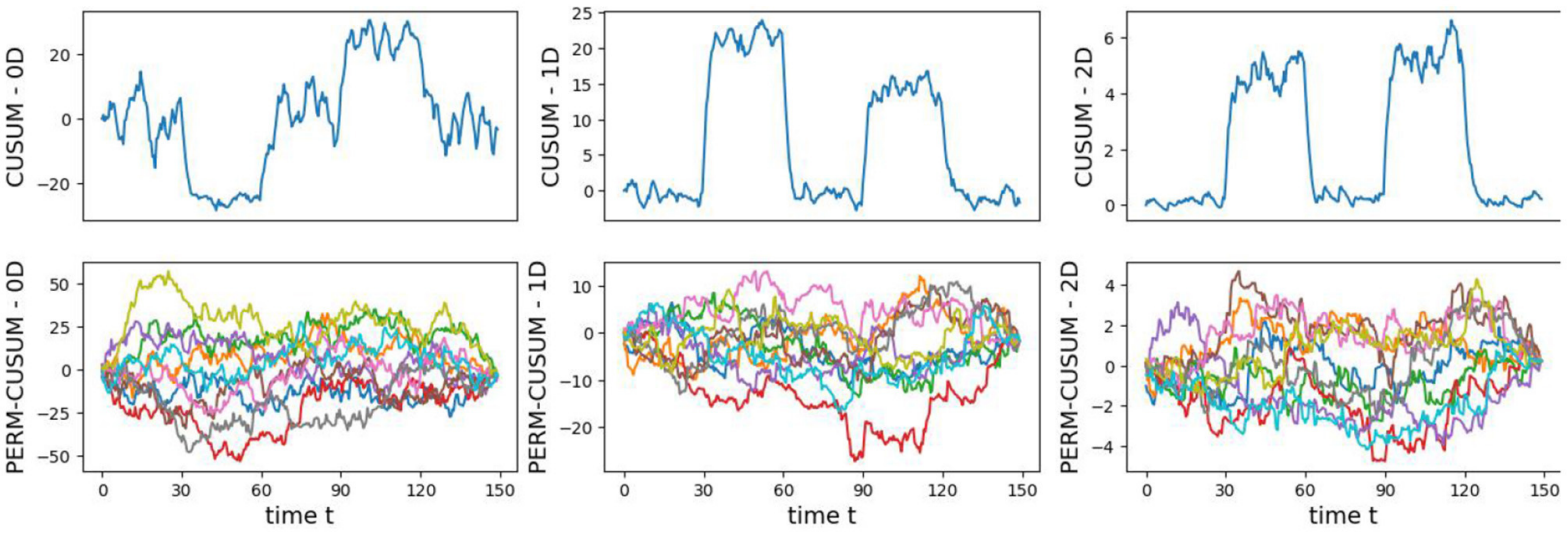
Observed CUSUM of deviations of total persistence, *S*_*k*_(*t*), and permuted CUSUM of deviations, $S_{k}^{*}\left(t\right)$.

**Figure 8:**
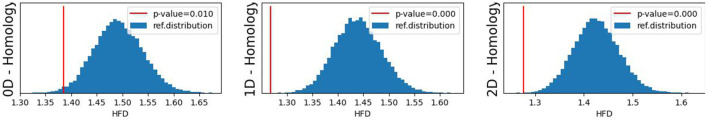
Observed *p*-values against reference distribution for the 0-, 1-, and 2-dimensional Homology groups.

The original CUSUM statistic as well as the permuted CUSUM statistics can be viewed in Figure 7. The *p*-values, showcased in Figure 8, provide valuable statistical insights into the estimated time-varying total persistence. The analysis notably reveals significant structural breaks in the second and third total persistence curves, indicating statistically meaningful changes in 1-, and 2-dimensional features over time. An increase in the total persistence of 1D and 2D-Homology suggests the emergence of holes and cavities in the cloud point, indicating a non-linear relationship among the components of the multivariate time series. Furthermore, the comparison of the three p-values indicates that alterations to 1D and 2D-Homology are much more significant than alterations to 0D-Homology. This comprehensive analysis confirms that the detected alterations in the topological patterns are not mere random fluctuations but rather statistically significant changes.

### 4.2 Second example: more complex patterns

**Figure 9:**
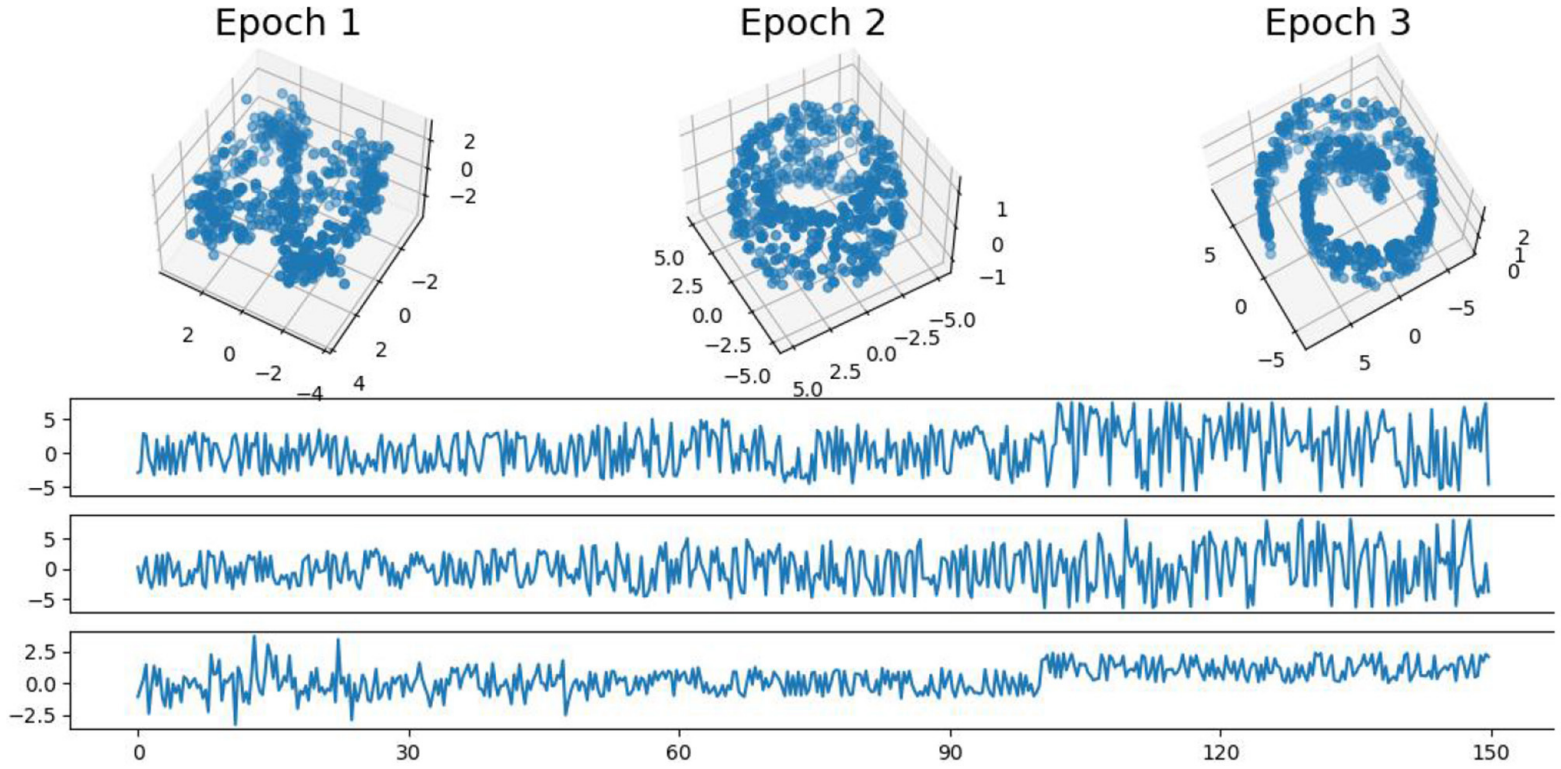
Simulated multivariate time series **(bottom)** with point cloud representation **(top)**. The first epoch exhibits an infinity-like pattern, the second epoch exhibits a torus-like pattern, and the third epoch exhibits a spiral-like pattern.

**Figure 10:**
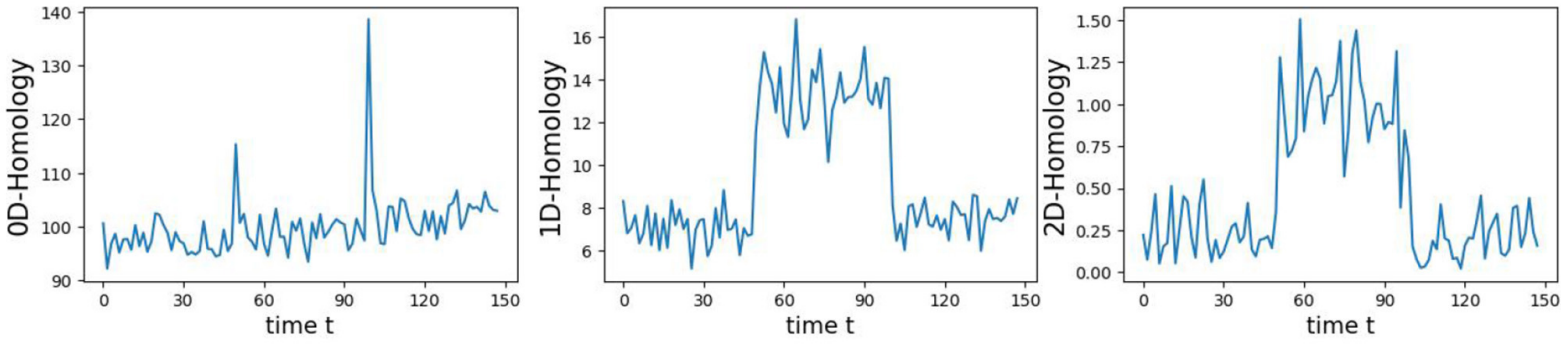
Estimated time-varying persistence curves reveal prominent peaks during the second (50–100 seconds) in both 1D and 2D-Homology, which is the expected distinction between torus and the other two patterns.

In this example, we generate a 3-dimensional time series consisting of 15,000 observations, equivalent to 150 seconds of data captured at a rate of 100 observations per second with more complex patterns (see Figure 9). During the first 50-second epoch (5,000 observations), the observations are drawn from an infinity-like pattern. In the second 50-second epoch (5,000 observations), the observations are drawn from a torus-like pattern. Finally, in the third 50-second epoch (5,000 observations), the observations are drawn from a spiral-like pattern. A visual representation of samples from this simulated data can be observed in Figure 9. Following the approach presented in Section 2, we employ a sliding window of size 150 and compute the total persistence to evaluate the dynamics of the topological patterns within the data. The estimated time-varying total persistence, illustrated in Figure 10, provides a clear depiction of the evolving topology over time. Notably, there is an increase in the total persistence of 1D and 2D homology, signifying the presence of larger holes/void in the point cloud during the second epoch (50–100 seconds). Conversely, a decrease in total persistence signals the disappearance of these topological patterns, leaving points randomly distributed in a spiral shape.

**Figure 11:**
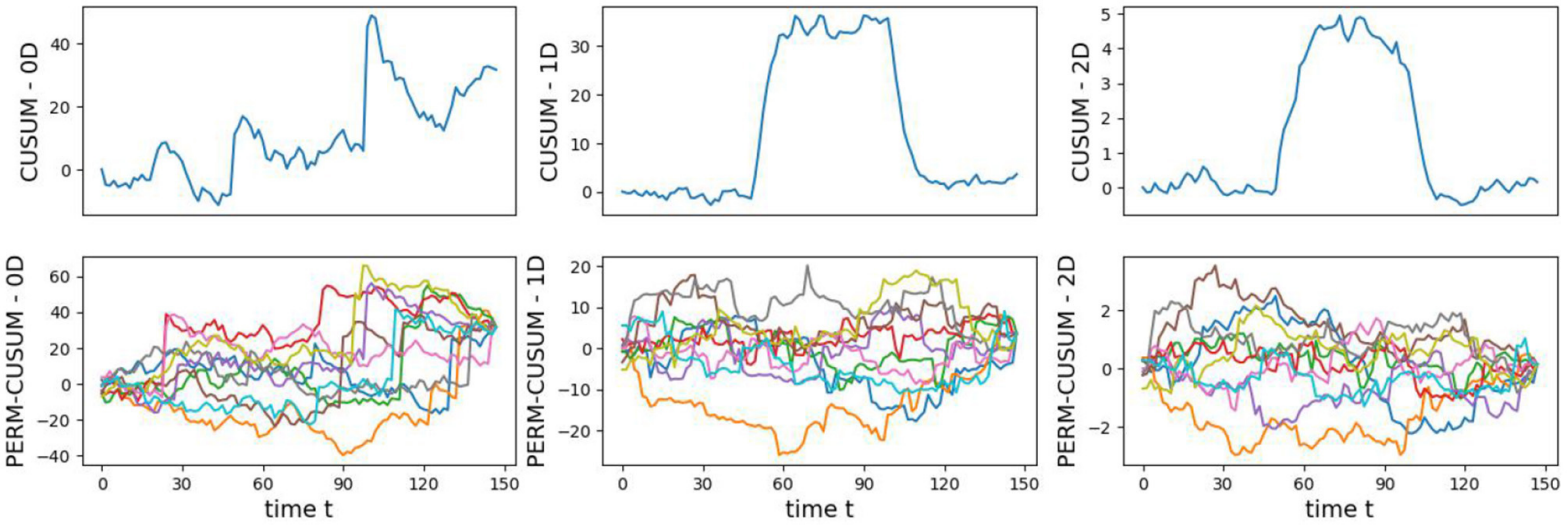
Observed CUSUM of deviations of total persistence, *S*_*k*_(*t*), and permuted CUSUM of deviations, $S_{k}^{*}\left(t\right)$.

**Figure 12:**
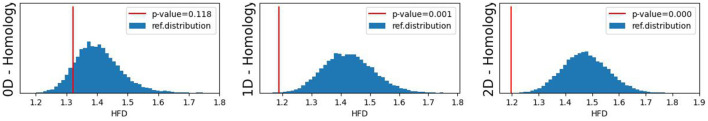
Observed *p*-values against reference distribution for the 0-, 1-, and 2-dimensional Homology groups.

The original CUSUM statistic as well as the permuted CUSUM statistics can be viewed in Figure 11. The *p*-values, showcased in Figure 12, provide valuable statistical insights into the estimated time-varying total persistence. The analysis notably reveals significant structural breaks in the second and third total persistence curves, indicating statistically meaningful changes in 1-, and 2-dimensional features over time. An increase in the total persistence of 1D and 2D-Homology suggests the emergence of holes and cavities in the cloud point, indicating a non-linear relationship among the components of the multivariate time series. Furthermore, the comparison of the three p-values indicates that alterations to 1D and 2D-Homology are much more significant than alterations to 0D-Homology. This comprehensive analysis confirms that the detected alterations in the topological patterns are not mere random fluctuations but rather statistically significant changes.

## 5 Application to epileptic seizure EEG signals

Epilepsy, a critical neurological condition affecting a significant portion of the population, manifests through abnormal neural firing during seizures. Initiated by a subpopulation of neurons, this abnormal activity can subsequently spread to other localized sub-populations or across the entire brain, giving rise to a variety of time-localized spikes and dynamic alterations in neural activity. In 2015, an estimated 3.4 million individuals in the United States, constituting around 1.2% of the total population, were affected by active epilepsy (Zack and Kobau, 2017). Despite its prevalence, epilepsy remains a complex disorder with limited treatment options (Chen et al., 2018). Therefore, understanding the underlying mechanisms and dynamics of epileptic seizures is crucial for improving diagnosis, treatment, and patient care.

Current data analytic methods in epilepsy research typically include high-dimensional parametric models (Rapela et al., 2019), stochastic differential equations (Tajmirriahi and Amini, 2021), spectral and coherence analysis (Busonera et al., 2018), and information theory (Stramaglia et al., 2021; Pernice et al., 2022). While these approaches have provided valuable insights, there is a growing recognition of the limitations they pose in capturing the intricate and often nonlinear relationships within neural networks during epileptic events. In light of these challenges, an emerging approach gaining attention is Topological Data Analysis (TDA). Unlike traditional methods, TDA offers a unique perspective by analyzing the shape and structure of complex data, allowing for a more holistic understanding of the underlying patterns in neural activity during seizures.

**Figure 13:**
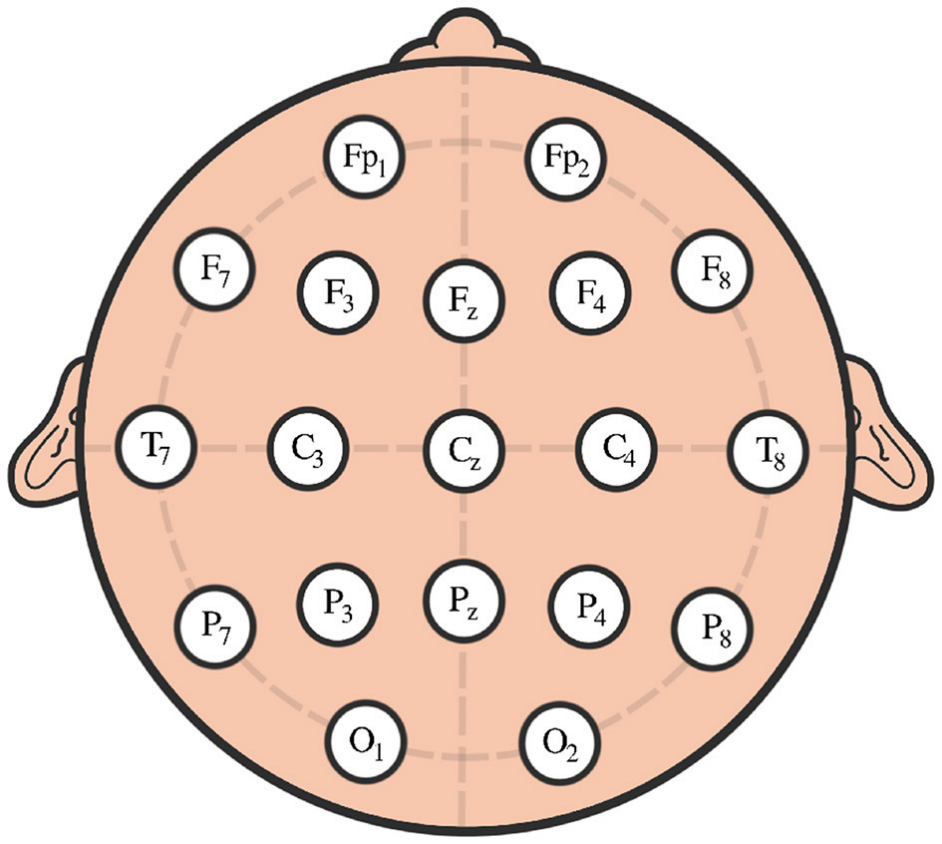
Scalp EEG with 10–20 standard layout.

**Figure 14:**
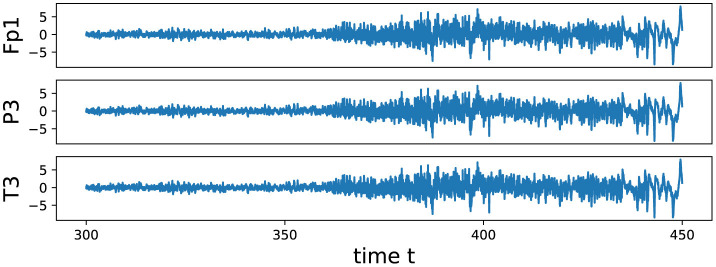
The figure displays 150 seconds of EEG data collected from the subset of Channels Left pre-frontal: Fp1; Left parietal: P3 and Left temporal: T3. The signals were sampled at a rate of 100 Hz. The data was recorded from a female patient diagnosed with left temporal lobe epilepsy. The dataset was collected by the Department of Neurology, University of Michigan.

**Figure 15:**
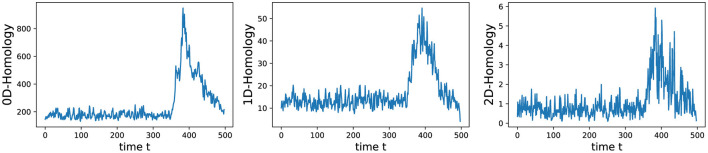
Estimated time-varying total persistence for 0-, 1- and 2-dimensional Homology groups.

**Figure 16:**
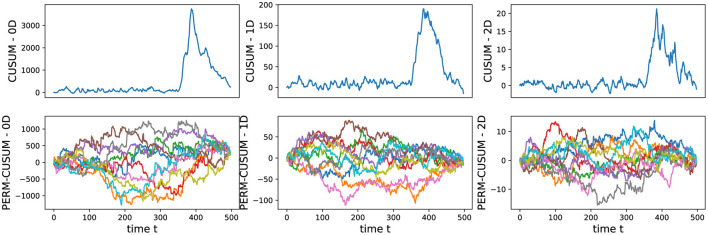
Visualization of the estimated **(top)** and permuted **(bottom)** cumulative sum of deviations for the 0-, 1-, and 2-dimensional Homology groups of total persistence.

**Figure 17:**
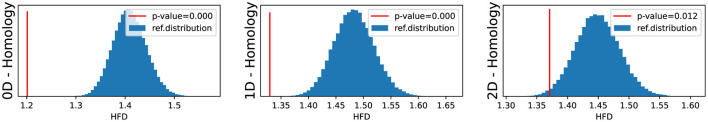
Fractal testing based *P*-Values for the 0-, 1-, and 2-dimensional Homology groups of total persistence.

We conducted an analysis of EEG signals recorded during an epileptic seizure from a female patient of Dr. Malow (formerly associated with the University of Michigan), diagnosed with left temporal lobe epilepsy (Ombao et al., 2001, 2005). The dataset comprises 19 bipolar scalp electrodes placed according to the 10–20 system, see Figure 13. Each recording spanning approximately 8 min and 20 seconds, sampled at a rate of 100 Hz. Figure 14 shows 3 of the 21 EEG signals (Left pre-frontal: Fp1; Left parietal: P3 and Left temporal: T3). The onset of the seizure episode was identified by the neurologist at around *t* = 363 seconds. The presence of non-stationarity, particularly amplitude variability, in the EEG signals during seizure motivated the use of our dynamic TDA approach. In Figures 15, 16, we report the time-varying total persistence curves as well as the estimated cumulative sum of deviations. Changes in the mean structure of these curves suggest the presence of dynamic topological patterns in the EEG signals during seizure time. Even these structural changes are convincing, at least from visual inspection, it is still necessary to formally assess their significance statistically. Therefore, following our approach in Section 3, we report the results in Figure 17. It is clear that all three curves display statistically extremely significant changes in their mean structure.

## 6 Conclusion

This paper presents a novel framework for analyzing and evaluating the significance of time-varying alterations of topological patterns by leveraging the concept of fractal dimension. Through a comprehensive simulation study, we have demonstrated the effectiveness of our approach in detecting substantial topological changes in localized point cloud embeddings. Additionally, its application to the analysis of EEG signals during epileptic seizures revealed noteworthy alterations in the dynamics of topological features, particularly in 0- and 1-dimensional Homology. These alterations, such as the increase in total persistence (1D-Homology) during epileptic seizures, suggest the emergence of holes in localized point cloud embeddings, signifying the development of a non-linear relationship among the components of the multivariate time series.

The versatility of our novel approach extends beyond EEG analysis, offering applicability to diverse settings for assessing structural breaks in measured time series. By introducing a robust testing framework and harnessing the power of topological data analysis, our methodology provides a valuable tool for comprehending and characterizing dynamic systems. Future research endeavors could explore its application to other domains, thereby enhancing our understanding of complex temporal phenomena and facilitating the development of targeted interventions across various applications.

## Data availability statement

The data analyzed in this study is subject to the following licenses/restrictions: none. Requests to access these datasets should be directed to HO, Hernando.Ombao@kaust.edu.sa.

## Ethics statement

Ethical approval was not required for the study involving humans in accordance with the local legislation and institutional requirements. Written informed consent to participate in this study was not required from the participants or the participants' legal guardians/next of kin in accordance with the national legislation and the institutional requirements.

## Author contributions

AE-Y: Conceptualization, Data curation, Formal analysis, Investigation, Methodology, Project administration, Software, Validation, Visualization, Writing – original draft, Writing – review & editing. MC: Funding acquisition, Resources, Supervision, Writing – review & editing, Conceptualization, Methodology. HO: Funding acquisition, Resources, Supervision, Project administration, Writing – review & editing.
